# Subcutaneous Implantable Cardioverter-Defibrillator Shock: Appropriate or Inappropriate?

**DOI:** 10.19102/icrm.2017.080504

**Published:** 2017-05-15

**Authors:** James E. Ip

**Affiliations:** ^1^Department of Medicine, Division of Cardiology, Cornell University Medical Center, New York, NY

**Keywords:** Implantable cardioverter-defibrillator shock, inappropriate shock, subcutaneous implantable cardioverter-defibrillator, T-wave oversensing, ventricular tachycardia

## Abstract

In this report, an unusual case of different defibrillator shocks in a single patient is described, and the interpretation and appropriate classification of subcutaneous implantable cardioverter-defibrillator therapy is discussed.

## Case presentation

**Figure 1:**
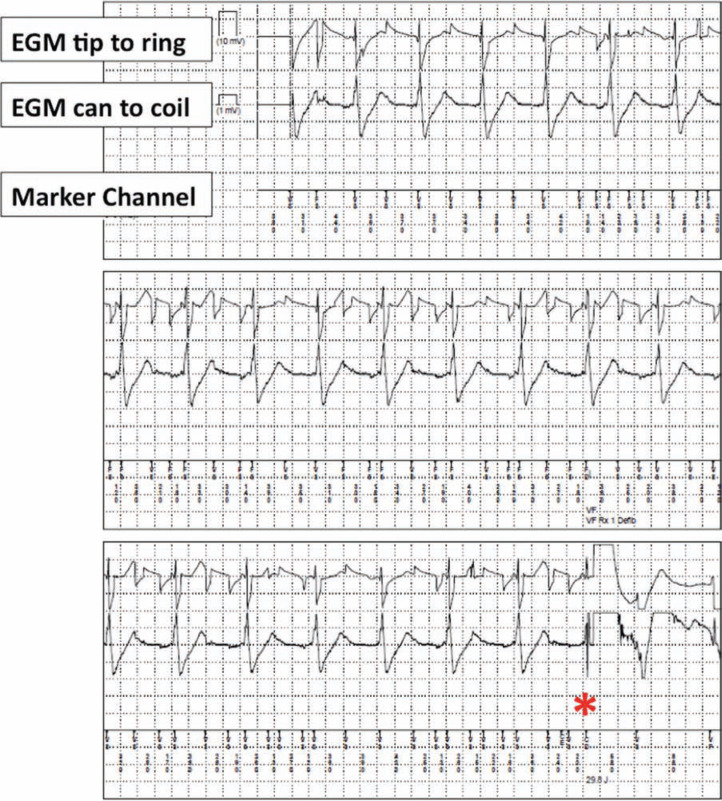
The device recording from the transvenous implantable cardioverter-defibrillator shows inappropriate detection from lead noise, shown on near-field electrogram and marker channel, which results in an ICD shock (denoted by the asterisk).

**Figure 2:**
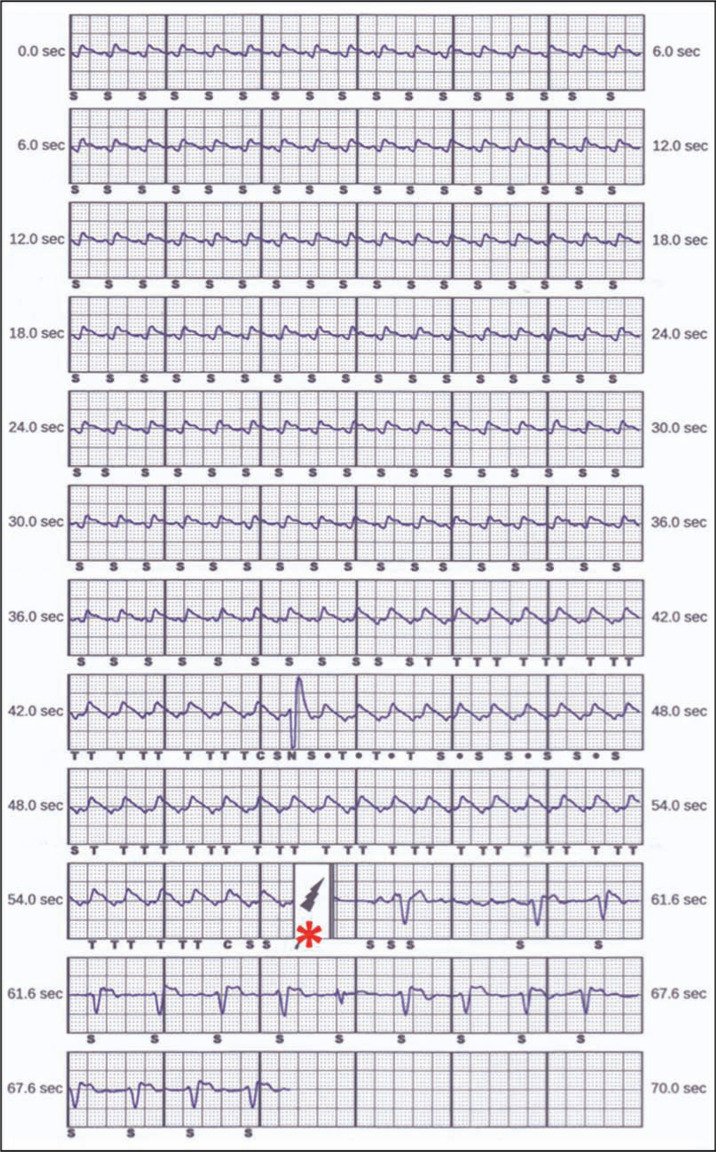
Device recording from the subcutaneous implantable cardioverter-defibrillator during presyncope that shows an S-ICD shock (denoted by the asterisk).

A 66-year-old woman with ischemic cardiomyopathy had a transvenous implantable cardioverter-defibrillator (ICD) implanted for primary prevention of sudden cardiac death. Nine years later, she suddenly received multiple ICD shocks. An interrogation of her device revealed a supraventricular tachycardia (SVT) at 240 bpm that resulted in an inappropriate shock. Moments later, lead noise resulted in several inappropriate shocks **(Figure 1)**, consistent with Riata™ (St. Jude Medical, St. Paul, MN, USA) ICD lead malfunction. During a lead revision procedure, she was found to have an occluded brachiocephalic vein. After a discussion of her options, she stated that she preferred to undergo subcutaneous ICD (S-ICD) placement, rather than lead extraction. The S-ICD was programmed with tachyarrhythmia therapies starting at 200 bpm, with a conditional zone up to 250 bpm for tachycardia discrimination. One year later, she received her first S-ICD shock, following an experience of sudden presyncope while supine **(Figure 2)**. However, it remained to be determined: was the shock appropriate?

## Discussion

**Figure 3:**
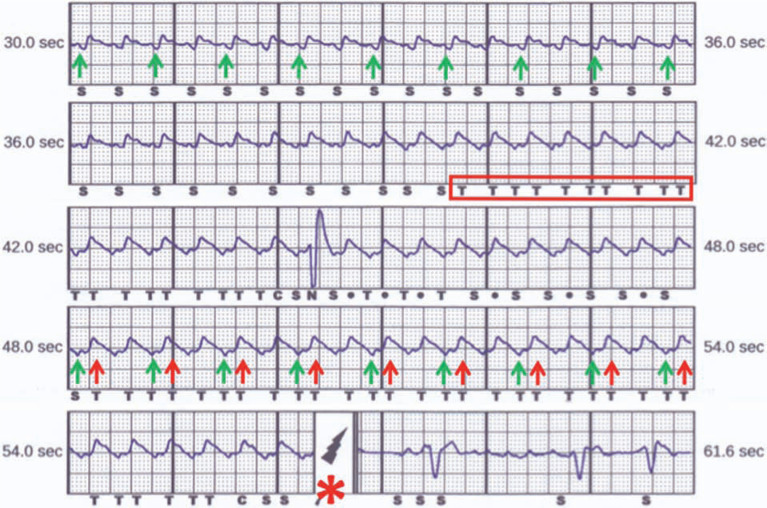
Device recording from the subcutaneous ICD during symptomatic monomorphic ventricular tachycardia shows that the device reclassified sensed events into the tachycardia zone (red box) when T-wave oversensing occurred. S, sense; T, tachycardia detection. The lightning bolt (asterisk) denotes the location where the shock is delivered. The green arrow shows QRS detection, while the red arrows show T-wave oversensing.

The device recording showed that the patient had ventricular tachycardia (VT) at approximately 170 bpm, a rate that is clearly slower than her detection zone. However, starting at 39.5 seconds into the recorded episode, there is double counting during the tachycardia, denoted by the “T” (tachycardia detection) markers. These detections are aligned with the QRS complexes (green arrows) as well as the T waves (red arrows), resulting in proper detection of VT **(Figure 3)**.

In comparison with transvenous ICD systems, in which tachyarrhythmia detection is initially based on timing analysis (i.e. rate, onset, stability, and atrioventricular relationship), after which morphology-based discrimination is applied, the S-ICD system initially analyzes the morphological characteristics of the electrogram recorded by the device to determine the presence of an arrhythmia.^1^ The S-ICD system evaluates up to 41 fiduciary points of each ventricular complex in order to accurately discriminate VT from SVT. The sensitivity of ventricular arrhythmia detection by S-ICD has been shown to be equivalent to transvenous ICD systems, and the specificity for SVT may be significantly better for the S-ICD system, especially when dual-zone programming (with a conditional zone) is applied.^2^ For arrhythmia discrimination, the signal is initially compared against a stored template for morphology comparison (static waveform analysis), followed by beat-to-beat variation during tachycardia (dynamic waveform analysis), and finally, QRS width is determined.

To prevent oversensing, the S-ICD sensing algorithm has a threshold that adapts to the amplitude of the R wave and decays over time. Although the signal is processed to prevent overdetection by double counting, T-wave oversensing (TWO) accounts for the majority of inappropriate shocks. In a pooled analysis of the IDE trial and EFFORTLESS registry, the incidence of inappropriate shock at three years was 11.7% with dual-zone programming, as compared with 20.5% with single-zone programming, and TWO accounted for 39% of all inappropriate shocks.^3^ Kooiman et al. found a 10.8% annual incidence rate of inappropriate shocks in their cohort, most commonly (73%) because of TWO during exercise or at elevated heart rates. They described the use of exercise testing to evaluate for TWO, and recommended selecting a more appropriate sensing vector and/or therapy zones by using a template acquired during exercise to avoid this undesirable outcome.^4^

While transvenous devices can also have TWO that may warrant another set of solutions,^5^ the S-ICD manufacturer recommends using a screening tool to exclude patients that have a high T-wave-to-QRS ratio during sinus rhythm. However, as shown in this case, our patient received appropriate therapy from this unintentional detection. Although TWO can often lead to inappropriate therapy, this recording shows the contrary—an inappropriate ventricular tachycardia detection that led to an appropriate S-ICD shock for symptomatic VT at a rate below the programmed detection zone.
